# Reviewing the History of HIV-1: Spread of Subtype B in the Americas

**DOI:** 10.1371/journal.pone.0027489

**Published:** 2011-11-23

**Authors:** Dennis Maletich Junqueira, Rúbia Marília de Medeiros, Maria Cristina Cotta Matte, Leonardo Augusto Luvison Araújo, Jose Artur Bogo Chies, Patricia Ashton-Prolla, Sabrina Esteves de Matos Almeida

**Affiliations:** 1 Programa de Pós-Graduação em Genética e Biologia Molecular, Universidade Federal do Rio Grande do Sul (UFRGS), Porto Alegre, Brazil; 2 Centro de Desenvolvimento Científico e Tecnológico (CDCT), Fundação Estadual de Produção e Pesquisa em Saúde (FEPPS), Porto Alegre, Brazil; 3 Laboratório de Medicina Genômica, Hospital de Clínicas de Porto Alegre (HCPA), Porto Alegre, Brazil; Institute of Infectious Disease and Molecular Medicine, South Africa

## Abstract

The dispersal of HIV-1 subtype B (HIV-1B) is a reflection of the movement of human populations in response to social, political, and geographical issues. The initial dissemination of HIV-1B outside Africa seems to have included the passive involvement of human populations from the Caribbean in spreading the virus to the United States. However, the exact pathways taken during the establishment of the pandemic in the Americas remain unclear. Here, we propose a geographical scenario for the dissemination of HIV-1B in the Americas, based on phylogenetic and genetic statistical analyses of 313 available sequences of the *pol* gene from 27 countries. Maximum likelihood and Bayesian inference methods were used to explore the phylogenetic relationships between HIV-1B sequences, and molecular variance estimates were analyzed to infer the genetic structure of the viral population. We found that the initial dissemination and subsequent spread of subtype B in the Americas occurred via a single introduction event in the Caribbean around 1964 (1950–1967). Phylogenetic trees present evidence of several primary outbreaks in countries in South America, directly seeded by the Caribbean epidemic. Cuba is an exception insofar as its epidemic seems to have been introduced from South America. One clade comprising isolates from different countries emerged in the most-derived branches, reflecting the intense circulation of the virus throughout the American continents. Statistical analysis supports the genetic compartmentalization of the virus among the Americas, with a close relationship between the South American and Caribbean epidemics. These findings reflect the complex establishment of the HIV-1B pandemic and contribute to our understanding between the migration process of human populations and virus diffusion.

## Introduction

The intense recent movements of human populations are reflected in the current diffusion and expansion of the HIV epidemic around the world [1]–[3]. Population bottlenecks, genetic recombination, genetic drift, and founder effects are characteristics associated with viral dissemination within these human populations and define the variability and nature of the establishment of the HIV/AIDS pandemic [4], [5]. A single transmission event in an unaffected area may result in the rapid spread of a unique viral form within a group with specific risk behaviors [6], [7], resulting in the establishment of the epidemic in that area [8], [9]. However, because of the rapid evolution of HIV and its global diffusion, the exact pathways of its dissemination are often unclear.

The emergence of HIV-1 resulted from the cross-species transmission of simian immunodeficiency viruses from chimpanzees to humans in West–Central Africa at the beginning of the 20th century [10]. Group M, responsible for the vast majority of HIV infections worldwide, initially spread throughout Africa, and in response to the actions of several genetic forces, has diversified into different subtypes [11]–[14]. The spread of these variants in the human population was not noticed for nearly eight decades. The first records of infection date from 1981 in American patients infected with subtype B viruses, who presented with clinical symptoms of what is today known as AIDS [15], [16].

The spread of subtype B from Africa initially occurred via a single introduction to Haiti in the 1960s, which was probably associated with the return of Haitian professionals from work missions in the Congo [5]. After the expansion of the epidemic in the Caribbean, current evidence points to the dissemination of the virus from there directly into North America. The subsequent transmission and spread of the virus in the United States allowed the epidemic to grow and expand to other parts of the world [5], [17]–[19]. Today, HIV-1 subtype B occupies an important position in the epidemiological profiles of various countries in Europe, Asia, and Africa, and is also the only subtype circulating in several countries in the Americas [19]–[23].

The dissemination of an infectious disease reflects the complex interactions between the infectious agent, its host, and the environment [24]. A strategy widely used in epidemiological research to identify the pathways of dissemination of an infectious agent is to combine the analysis of sociodemographic evidence with that of complementary phylogenetic data [3], [5], [24], [25]. In a recent study of the evolutionary history of HIV-1 subtype B, Gilbert et al. (2007) demonstrates the emergence of this subtype from Africa to American countries (starting in Haiti) by examining 117 subtype B sequences from 19 countries [5]. However, South America was poorly represented in this work, with only nine sequences from four countries. Considering that the Caribbean has close economic, historical, and even social relationships with several countries in South America [26]–[28], it is reasonable to investigate if HIV-1B was directly transmitted from the Caribbean to South American countries. Thus, the present study aimed to investigate by phylogenetic and genetic statistics analyses the role of South American countries in the establishment of the HIV-1 subtype B in the Americas.

## Materials and Methods

### Dataset Selection

Around 6000 HIV-1 subtype B sequences of protease and portions of reverse transcriptase segments of the *pol* gene (nucleotides 2253–3233 relative to strain HXB2) were selected from the Los Alamos HIV Sequence Database (http://www.hiv.lanl.gov/) and GenBank (http://www.ncbi.nlm.nih.gov/nucleotide/). To ensure the selection of high-quality data, we selected the sequences that met the following criteria: (a) the samples were isolated from patients living in the Americas; (b) the country of origin was clearly established; (c) only one sequence per patient was included; (d) no report of intersubtype recombination; (e) no evidence of hypermutation; and (f) no occurrence of premature stop codons, frameshift mutations, or ambiguity saturation (excess of undetermined nucleotides). Moreover, sequences from the same country of isolation, describe in the same study and phylogenetically close related were excluded from our dataset. To understand the spread of the virus without compromising the quality of the results, all the sequences were examined for evidence of intersubtype recombination. We selected sequences with a minimum confidence threshold for pure subtype B of 0.95 with a window size of 200 nt, using the RIP tool at the Los Alamos HIV Sequence Database. The dataset was also evaluated using additional reference sequences by constructing a neighbor-joining phylogeny, to guarantee the selection of nonintersubtype recombinants. Although all HIV sequences currently described can be considered intrasubtype recombinants at some level, these evolutionary events are probably insignificant in the context of the origin and geographical grouping of HIV subtypes [29], [30]. After a carefully selection, a dataset of 313 HIV-1B *pol* publically available sequences retrieved from 27 countries from North America, South America, Central America and Caribbean were used in the following analyses.

Sequence alignments were created using MUSCLE [31] and manually edited to optimize them. Four African sequences of subtype D and two of subtype C were selected as outgroups. Three different alignments were constructed for this study: one set including 313 sequences for ML analysis, one set comprising 263 sequences for Bayesian analysis, and a third set for the genetic structure analysis. These sets are available upon request.

### Phylogenetic Reconstruction

The reconstruction of phylogenetic trees was performed with the maximum likelihood (ML) method using a set of 313 subtype B sequences that met our quality criteria (Table S1). The ML analysis was conducted with the program phyML [32] under the GTR model of nucleotide substitution, with a proportion of invariable sites, and substitution rate heterogeneity (GTR+G+I). Nearest-neighbor interchange was used for heuristic tree searches. Support for the internal nodes was obtained with parametric bootstrapping using 1000 replicates.

A Bayesian Markov Chain Monte Carlo (MCMC) approach, implemented in BEAST ver. 1.5.4 [33], was used with a set of 263 sequences (Table S1) to reconstruct the phylogenetic tree and estimate the date of the most-recent common ancestor of the epidemic in the Americas. The evolutionary history was inferred with a Bayesian Skyline (BSP) coalescent tree prior, under an uncorrelated lognormal relaxed clock, and the GTR+I+G model of nucleotide substitution. Three independent runs of 300 million steps sampled every 30,000 generations were performed and the effective sample size was evaluated in TRACER [34]. The maximum sum of clade credibility tree was selected from the posterior tree distribution.

We inferred an ML phylogeny to investigate the role of South America in the HIV subtype B epidemic. This approach supported a relationship between the sequences from the Caribbean and those from South America. Notably, the sequences from Colombia, Venezuela, Brazil, Suriname, and Guyana were intermingled with those from Trinidad and Tobago, the Dominican Republic, and Haiti.

To further explore these relationships, Bayesian trees were inferred using 263 sequences (Table S1) representing North America (n = 71), Central America and the Caribbean (n = 88), and South America (n = 104) under an uncorrelated relaxed clock and with a Bayesian Skyline coalescent tree prior. The effective sample size (ESS) was calculated by combining the outputs from the three runs for each model, and excluding the first 10% of steps as the burn-in for each chain. The Bayesian MCMC-independent runs converged on similar values and all parameter estimates showed ESS values of more than 200.

### Genetic Diversity

The population genetic structure of HIV subtype B among the countries of North America, Central America, South America, and the Caribbean was quantified using estimates of the F statistics [35]. A set of 308 sequences was built, excluding countries represented by only one sequence, and any ambiguous nucleotide was changed to “N”. Estimates were calculated using analysis of molecular variance (AMOVA) [36] in Arlequin ver. 3.5.1.2 [37] under the Kimura two-parameter model with 10,000 randomizations. Invariable sites were included and sites with gaps/missing data were considered. A nonmetric multidimensional scaling plot was obtained with SPSS ver. 8 (Inc., Chicago, IL).

## Results

### Phylogenetic Analysis

**Figure 1:**
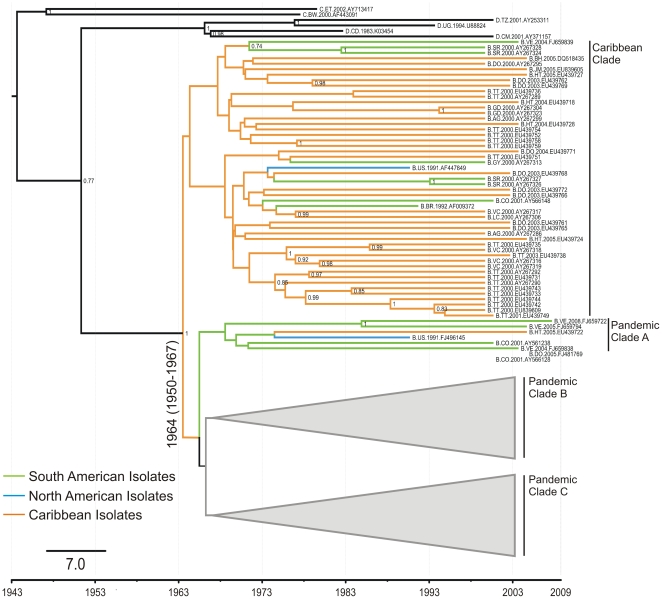
Bayesian tree of 263 HIV-1 subtype B sequences of the pol gene from 25 American countries. Majority-rule Bayesian consensus tree of 268 HIV-1 subtype B *pol* sequences isolated in 25 countries in the Americas. The outgroups are subtypes C and D. Branches are colored according to the sample origin. Orange branches represent isolates from the Caribbean, the green branches represent South American isolates, and the blue branches represent isolates from North America. Posterior probabilities are shown for the key nodes. The tips of the tree contain isolate information regarding the subtype, country, year of isolation, and GenBank accession number. Abbreviations of countries are as follows: AR, Argentina; AG, Antigua and Barbuda; BH, Bahamas; BR, Brazil; BW, Botswana; CA, Canada; CD, Democratic Congo; CM, Cameroon; CO, Colombia; CU, Cuba; DO, Dominican Republic; EC, Ecuador; ET, Ethiopia; GD, Grenada; GY, Guyana; HT, Haiti; JM, Jamaica; LC, Santa Lucia; SR, Suriname; TT, Trinidad and Tobago; TZ, Tanzania; UG, Uganda; US, United States; VC, St Vincent; VE, Venezuela.

The Bayesian genealogies have topologies similar to the ML trees, supporting an older clade that includes isolates from different countries in the Caribbean (Figure 1). The sequences from Haiti, the Dominican Republic, Trinidad and Tobago, Santa Lucia, and St Vincent are nested together in these deep branches. Interestingly, eight isolates from South American countries, including Suriname, Guyana, Brazil, Colombia, and Venezuela, and one from the United States are intermingled within this clade. Apart from the two clusters formed by the Trinidad and Tobago isolates, no other country showed a compartmentalized grouping of their sequences. The finding that 52% of the HIV-1B Caribbean sequences could be traced back to a unique most-recent common ancestor suggests a single major introduction event of HIV-1B from Africa, followed by its local spread (Table S2). Using an evolutionary time scale spanning 26 years, the Bayesian analysis indicates that the HIV-1B epidemic in the Caribbean countries evolved from a common ancestor introduced around 1964 (1950–1967).

**Figure 2:**
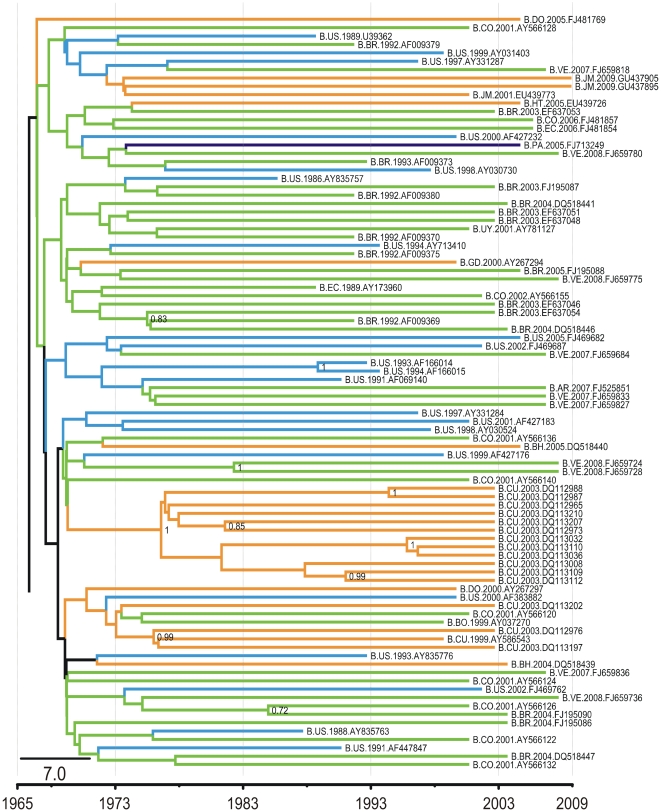
Part of the Bayesian tree of 263 HIV-1 subtype B sequences of the pol gene (Pandemic Clade B). This is the full version of the collapsed clade presented in the Figure 1. The orange branches represent the isolates from the Caribbean, the purple branches represent Central American isolates, the green branches represent the South American isolates, and the blue branches represent the isolates from North America. Abbreviations of countries are as follows: AR, Argentina; AG, Antigua and Barbuda; BH, Bahamas; BR, Brazil; CA, Canada; CO, Colombia; CU, Cuba; EC, Ecuador; HN, Honduras; HT, Haiti; JM, Jamaica; MX, Mexico; PA, Panama; PE, Peru; TT, Trinidad and Tobago; US, United States; UY, Uruguay; VE, Venezuela.

From the same common ancestor of the Caribbean Clade, there arose three clusters in which sequences from North America, Central America, South America, and the Caribbean are intermingled (Pandemic Clades). The small cluster (pandemic clade A, Figure 1) positioned basal to the pandemic clades is composed of six isolates, including three from Venezuela and one from Colombia, indicating that the initial dispersion of subtype B in the Americas seeded the Caribbean epidemic and that in nearby countries. Those sequences that occupy more derived positions are grouped into two clusters (pandemic clade B and pandemic clade C) that share a common ancestor with pandemic clade A. Pandemic clade B (Figure 2) is mainly composed of South American isolates (48%; Table S2), including 41% of the total sequences from South American countries. Within this clade, the sequences positioned nearest the tree root also derive from South American countries, suggesting that this clade originated in that region. The Brazilian isolates form a monophyletic cluster that seems to represent the main evolutionary history of the pandemic in that country because only four Brazilian sequences cluster outside this clade. Similarly, 12 isolates from Cuba cluster together with sequences from South America, indicating that the Cuban epidemic originated from South American countries.

**Figure 3:**
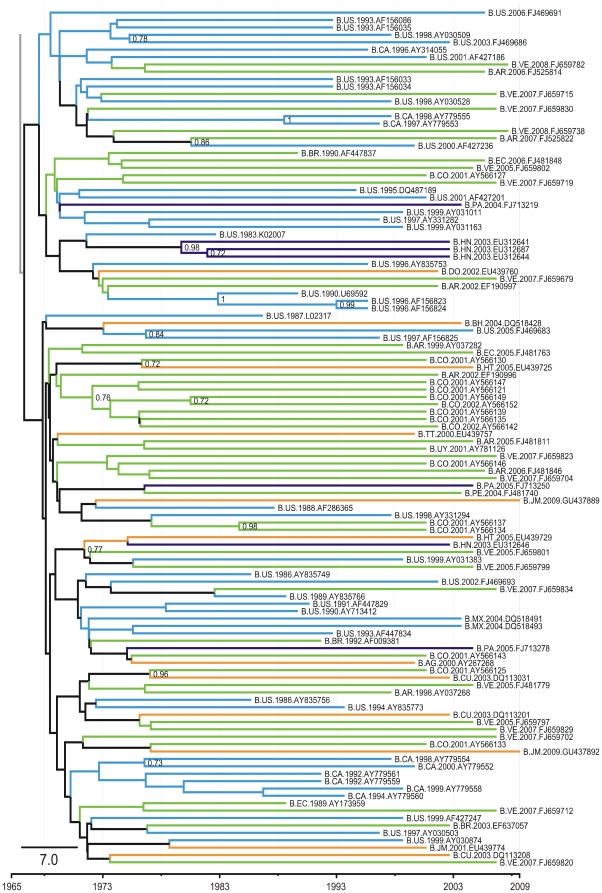
Part of the Bayesian tree of 263 HIV-1 subtype B sequences of the pol gene (Pandemic Clade C). Part of the 50% majority rule consensus tree constructed from the Bayesian MCMC (BEAST) analysis. This is the full version of the collapsed group shown in Figure 1, indicating the evolutionary relationships among the sequences in the pandemic clade. The orange branches represent the isolates from the Caribbean, purple branches represent isolates from Central America, the green branches represent the South American isolates, and the blue branches represent the isolates from North America. The branches are not drawn to scale. Abbreviations of countries are as follows: AR, Argentina; AG, Antigua and Barbuda; BH, Bahamas; BR, Brazil; BW, Botswana; CA, Canada; CD, Democratic Congo; CM, Cameroon; CO, Colombia; CU, Cuba; DO, Dominican Republic; EC, Ecuador; ET, Ethiopia; GD, Grenada; GY, Guyana; HT, Haiti; JM, Jamaica; LC, Santa Lucia; SR, Suriname; TT, Trinidad and Tobago; TZ, Tanzania; UG, Uganda; US, United States; VC, St Vincent; VE, Venezuela.

The grouping of South American sequences with North American isolates, mainly those from the United States, within pandemic cluster B in recent times (on the evolutionary time scale) could indicate ongoing viral gene flow between the two Americas in both directions. The existence of several independent South American clades also indicates distinct transmission networks, originating from different introduction events at different time points (Figure 3).

### Genetic Diversity Analysis

We used a statistical genetic framework to understand the relationships between the epidemics of HIV-1 subtype B in North America, South America, and the Caribbean. Differences in the degree of genetic diversity among the continents was calculated using Φ_ST_ estimates, providing further evidence for the genetic structure of the HIV-1 subtype B population of the Americas (Table 1 and Figure 2). The highest level of viral molecular variation among the continents suggests a separation between the Caribbean and North America (Φ_ST_: 0.04373, P<0.00001). Despite the genetic structure among the three regions, estimates of Φ_ST_ values indicate a closer relationship between the Caribbean and South America viral strains (Φ_ST_: 0.03022, P<0.00001; Table 1).

**Table 1:** Analysis of molecular variance (AMOVA) of HIV-1 subtype B isolates from North, Central, and South America.

Division (number of populations tested)	Source of Variation	Fixation index
North America (3) *vs.* Central America (11) *vs.* South America (8)	Among divisions	Φ*ct:* 0.00663
	Among populations within divisions	Φ*sc:* 0.06225*
	Within populations	Φ_ST_:0.06847*
North America *vs.* Central America *vs.* South America	Among divisions	Φst: 0.02457*

*p<0.05.

**Figure 4:**
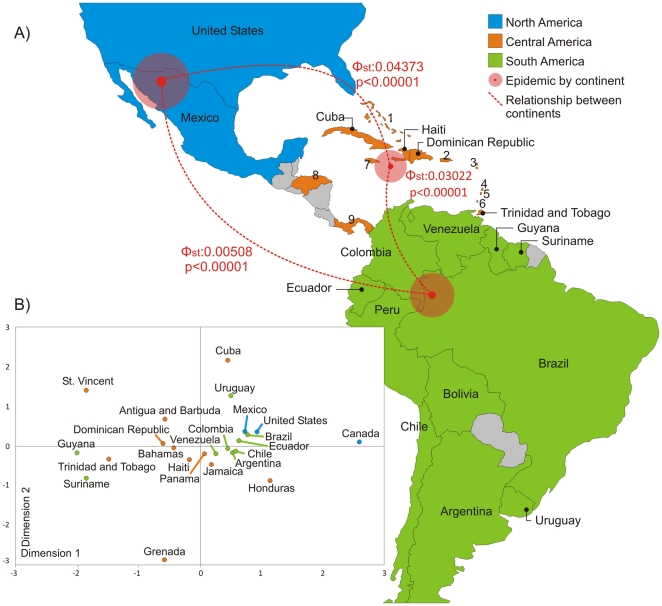
Genetic Structure of 308 HIV-1 subtype B sequences from American Countries. Synthetic map illustrating the distributions and geographic origins of strains isolated in the Americas and the genetic structure among continents and countries. (**a**) Countries of sample isolation are colored according to geopolitical regions, comprising South America, Central America (including the Caribbean), and North America. No isolates from the gray-colored countries were included in this study. Countries located in Central America are represented by numbers: (1) Bahamas, (2) Puerto Rico, (3) Antigua and Barbuda (4) Santa Lucia, (5) St Vincent, (6) Grenada, (7) Jamaica, (8) Honduras, and (9) Panama. The red dotted lines represent Φ_ST_ estimates between continents. South American sequences are genetically intermediate between those of Central America and North America. (**b**) Nonmetric multidimensional scaling plot of the Φ_ST_ estimates among 22 South American countries. Dimension 1 separates the 308 isolates by country.

We also constructed a multidimensional scaling plot based on Φ_ST_ for sequences from 22 countries (Figure 4). Those from South American countries grouped within a cluster of North America sequences and a cluster primarily composed of sequences from Caribbean countries (Figure 4). It is interesting to note that the samples from South American countries, with the exception of Guyana and Suriname, were more tightly clustered than the isolates from the Caribbean or North America. The samples from Guyana and Suriname grouped together with those from Trinidad and Tobago in the Caribbean cluster. The viruses from Mexico and the United States seem to be genetically related to the epidemics in Brazil and other South American countries. The Canadian sequences maintain a close relationship with those of the United States, despite Canada's position distant from the other countries of North America.

## Discussion

The emergence and dispersal patterns of HIV-1 subtype B from its epicenter in Africa were major events in the history of the epidemic, which has become a major public health issue. The HIV-1 subtype B pandemic has been the focus of several research groups in distinct disciplines, because it was the first subtype to be isolated in industrialized nations and the first to spread with mobile populations [17], [38]–[40]. In the field of phylogeography, HIV-1 subtype B is the subject of continuing debate. Several suppositions have been made about its temporal and geographical distribution patterns, including its origin and dissemination [5], [41]–[43]. Gilbert et al. (2007) fueled the discussions when they traced the initial spread of the epidemic from Africa in 1966 (1962–1970), showing that the epidemic of subtype B most likely began in Haiti, given the monophyletic cluster of sequences from this nation [5]. The authors suggested that Haiti was the key conduit for the introduction of subtype B into the United States before its global dissemination. Our results add another piece to this epidemiological puzzle, providing evidence that the spread of HIV-1B in the early 1960s in the Americas was not as unidirectional as initially suggested. The phylogenetic and statistical approaches used here point to the significant participation of South American countries in the transmission and evolution of the HIV-1 subtype B epidemic in the Americas.

The Caribbean countries undoubtedly played an important role in the HIV-1 subtype B epidemic. On the *pol* gene phylogeographic reconstruction, a consistent clade of isolates from the Caribbean arose simultaneously from the same common ancestor of the Pandemic Clade (A+B+C). Although the results inferred from our phylogenetic trees do not elucidate the basal position of the Caribbean Clade with respect to the Pandemic Clade, our analysis of genetic diversity and the results of previous studies that included ancestral sequences point to this conclusion [5], [41]. In addition, we cannot state that Haiti [5] or any other country was the origin of the subsequent dissemination because ancient sequences are unavailable. Within the Caribbean clade, the epidemic in Trinidad and Tobago seems to have primarily derived from two or more effective HIV-1 introduction events, because on all the trees obtained, most of the sequences from this country form two distinct clades (Figure 1). This result differs from the findings of previous studies that, based on the *gag* and *env* genes, and on the nearly complete HIV-1 genome, reported a monophyletic epidemic in that country [5], [19], [44]. We also provide evidence for the introduction of non-pandemic subtype B clades from the Caribbean into countries in northern South America, such as Suriname, Guyana, Brazil, Colombia, and Venezuela. Despite the existence of phylogenetic evidence that the United States was the midpoint between Caribbean countries and the global spread of HIV-1 subtype B, our results do not show a direct transmission event of the HIV-1B from the Caribbean to the United States early in the epidemic. The direct introduction of the virus into the United States from the Caribbean would have generated a genetic signature in the viral genome, and when dealing with appropriate genetic markers, such as the *pol* gene, the phylogenetic pattern expected under such model should effectively group a significant number of sequences from the United States, dating from the beginning of the epidemic, within the Caribbean strains. Our evolutionary analysis of HIV-1B agrees with other studies in the timing of its introduction into the Americas [5], and estimates the date of the most-recent common ancestor to be 1964 (1950–1967). The clustering of the Caribbean strains together in older branches could be the result of founder effects from one or a few introductions to that region in the 1960s.

Both the Bayesian and ML methods show a cluster derived from the same common ancestor of the Caribbean clade that groups isolates collected at different times from South American countries (pandemic clade A), albeit with low probability. This cluster occupies a position basal to the pandemic clades within subtype B (Figure 1), providing evidence for two epidemiological scenarios: (a) the direct introduction of HIV-1B into South America, which seeded a secondary outbreak in the United States; or (b) the concurrent spread of HIV-1B from the Caribbean to South America and North America.

Supporting scenario a, the beginning of the HIV epidemic in the Americas coincided with a boom in oil production by Venezuela [26], [28]. Great changes in its economic situation caused Venezuela to implement policies to attract immigrants from Colombia, Ecuador, Peru, Cuba, Trinidad and Tobago, the Dominican Republic, and the United States [26], [28]. The population of migrants from other Latin American countries tripled between 1970 and 1980 according to Venezuela's censuses [26]. It would not be surprising if this movement of people from the Caribbean also introduced and disseminated the HIV epidemic into South America. Further reinforcing our hypothesis, Leal and VillaNova (2010) used 66 near-full-length genomic sequences (8160 bp) of worldwide HIV-1 subtype B isolates to show that the epidemic in Brazil, a South American country, shares a common ancestor with a “North American–European cluster”, and that Haitian strains occupy the deepest positions in this phylogeny [44]. Together, these various lines of evidence suggest a link between the Caribbean epidemic and the direct introduction of HIV-1B into South America.

However, according to scenario a, after spreading to the countries of South America, the virus was introduced from there into North America. Emigrations from Mexico to the United States have been the largest migratory movements on the planet [26]. Therefore, Mexico could represent the “entrance door” for the epidemic into the United States. Interestingly, of the 10 phylogenetic relationships involving Mexican sequences throughout the whole ML tree, eight are linked to South American strains (80%). The Bayesian trees include only two Mexican sequences because information about the sampling dates was unreliable (Table S1).

Finally, the bidirectional spread of HIV-1B from the Caribbean, as suggested by scenario b, may also have participated in the establishment of the epidemic in the Americas because three distinct clusters, including pandemic cluster B, arise from the Caribbean clade on the Bayesian trees. The historical factors involved in scenario a could also support scenario b. Furthermore, the historical registers of AIDS cases among Haitian individuals in the 1970s in the United States are an indication of the pathway of the epidemic into North America from the Caribbean [45]. It is also possible that the HIV-1B pandemic in the Americas arose from both scenarios acting simultaneously. In this case, the epidemic in the South American countries might have originated from the virus circulating in the Caribbean and from the diffusion of the pandemic from the United States.

The epidemic in Cuba deserves special attention because in addition to its extraordinary genetic diversity [46], Cuba seems to have a remarkable subtype B epidemic. One clade encompassing 12 Cuban sequences is clearly related to samples from South American countries, suggesting a different epidemiological link to that inferred for the Caribbean region. Such a relationship supports the hypothesis that the expansion of the epidemic from the Caribbean countries was not specifically directed towards the United States, as previously suggested [5]. After all, South American countries played an important role historically in the definition of the politics of Cuba [47].

As well as the phylogenetic analysis, we calculated the degree of differentiation between the continent-specific compartments of the epidemic using AMOVA. Human migrations are a confounding factor in the already complex dissemination of HIV. The exchange of people among countries can mix viral subpopulations and mask the historical processes that create patterns of genetic variation and geographic signatures within an epidemic [24]. Despite the high rate of migration in the Americas, the data presented here demonstrate a significant degree of viral population structure within the various regions (Table 1 and Figure 4). Such structuring can originate in the selection effects of the host's immune system, in recombination processes, and in founder effects [19]. However, there is a lack of evidence of such selection [19] and all the sequences analyzed here met the criterion of no intersubtype genetic mixing. Therefore, founder effects that acted on the viral population in the beginning of the epidemic and are still detectable 40 years later seem to better explain the genetic compartmentalization observed.

The Φ_ST_ estimates, which test the viral population structure in the Caribbean, North America, and South America, show that the Caribbean epidemic has a closer genetic relationship to that established in South America (Figure 2), suggesting an epidemiological link between the two Americas. However, the Φ_ST_ estimates from the South American and North American epidemics point to the circulation of genetically related viruses, attributable to the constant movement of human populations between these two regions. The relationships among the sequences from specific countries are shown in Figure 2 and corroborate some of the results derived from our trees. Again, it seems that the Caribbean strains are more closely genetically related to those from South American countries than to those from North America. Furthermore, apart from Guyana and Suriname, which have epidemics related to that of Trinidad and Tobago, the sequences from all the countries of South America are closely related, suggesting an intricate epidemic for that continent. Finally, the role of Mexico in the dispersal of HIV-1B throughout the Americas should be rethought, because our phylogenetic and genetic statistical analyses point to a connection between the United States, Mexico, and the South American countries.

The low support displayed in our Bayesian tree is probably linked to the extreme genetic conservation of the pol gene, combined with the large number of sequences used in this study. We recognize that the *pol* gene may not offer sufficient genetic variation to ensure a strong phylogenetic signal and thus confer sufficient statistical support for the branches of our trees. However, a recent study evaluating the evolution of bacterial genes under simulated biological conditions revealed that realistic estimates of the statistics not necessarily estimate how well a reconstructed phylogeny that actually represents cladistic relationships exist in nature [48]. Moreover, our analysis successfully reiterated the date and geographic trace of previous studies based on other genes, such as *env* and *gag* [5], [19], [44]. Similarly, it was demonstrated that this gene is useful in the identification of transmission events by phylogenetic means even when codon positions associated with drug resistance are maintained [49]. We used the *pol* gene because it provided the largest set of dated sequences, sampled across the widest possible number of countries in the Americas. The recovery of ancestral sequences from American countries, especially South American countries, should undoubtedly better trace the spread of HIV-1B, strengthening the support for one or other possible scenario.

Our results do not contradict those of previous studies, but in fact, our statistical genetic and phylogenetic analyses add further pieces to the historical puzzle of HIV-1 subtype B in the Americas, revealing that part of the epidemic in South America derived directly from the Caribbean epidemic. We propose a scenario that began with the introduction and spread of the virus locally in the Caribbean region, followed by its dispersal into northern South America, establishing an epidemic genetically similar to that in the Caribbean. An epidemiological link between South America and North America was easily established by several waves of migration from the various countries of Latin America to the United States [26]. However, a direct link between the Caribbean and North America also contributed to the dissemination of HIV-1B and historical registers confirm this connection [50], [51]. The data presented here offers a new perspective on the epidemic of HIV-1 subtype B in the Americas. This work also highlights the utility of population genetic methods in understanding the evolution and spread of this epidemic, contributing primarily to our understanding of the interactions between the virus and the migration processes governing the diffusion of human populations.

## Supporting Information

Table S1 Description of the geographic origin and year of sampling of 313 HIV-1 subtype B sequences retrieved from the Los Alamos HIV Sequence Database used to infer the pathways of dissemination of subtype B through the Americas.
(DOC)

Table S2 Percentage of sequences grouped within each of the four main clades inferred in the Bayesian phylogeny of HIV-1 subtype B using 263 sequences from 25 countries sampled in North America, Central America, Caribbean and South America.
(DOC)
