# Supplementary material for: Reviewing the History of HIV-1: Spread of Subtype B in the Americas
Source: PLoS One. 2011 Nov 23;6(11):e27489. doi: 10.1371/journal.pone.0027489 (PMC3223166; doi:10.1371/journal.pone.0027489)
Supplement: Table S1 — Description of the geographic origin and year of sampling of 313 HIV-1 subtype B sequences retrieved from the Los Alamos HIV Sequence Database used to infer the pathways of dissemination of subtype B through the Americas. (DOC) [file pone.0027489.s001.doc]

**Table S1.** Geographic origins, time spans, and numbers of sequences used to trace the dissemination of HIV-1 subtype B in the Americas.

| **Region** | | **Country** | **Country Code** | **Number of Sequences** | | **Total** | **Time frame** |
| --- | --- | --- | --- | --- | --- | --- | --- |
|  |  |  |  | dated | undated |  |  |
| 1. | Caribbean | Antigua and Barbuda | AG | 3 | 2 | 5 | 2000 |
| 2. | Caribbean | Bahamas | BH | 4 | - | 4 | 2004-2005 |
| 3. | Caribbean | Cuba | CU | 19 | - | 19 | 1999-2003 |
| 4. | Caribbean | Dominican Republic | DO | 12 | - | 12 | 2000-2005 |
| 5. | Caribbean | Grenada | GD | 3 | - | 3 | 2000 |
| 6. | Caribbean | Haiti | HT | 8 | - | 8 | 2004-2005 |
| 7. | Caribbean | Jamaica | JM | 7 | - | 7 | 2001-2009 |
| 8. | Caribbean | Santa Lucia | LC | 1 | - | 1 | 2000 |
| 9. | Caribbean | Puerto Rico | PR | - | 1 | 1 | - |
| 10. | Caribbean | Trinidad and Tobago | TT | 19 | - | 19 | 2000-2001 |
| 11. | Caribbean | St. Vincent | VC | 4 | - | 4 | 2000 |
| 12. | Central America | Honduras | HN | 4 | - | 4 | 2003 |
| 13. | Central America | Panama | PA | 4 | - | 4 | 2004-2005 |
| 14. | North America | Canada | CA | 9 | - | 9 | 1992-2000 |
| 15. | North America | Mexico | MX | 2 | 8 | 10 | 2004 |
| 16. | North America | United States | US | 60 | - | 60 | 1983-2006 |
| 17. | South America | Argentina | AR | 9 | 4 | 13 | 1998-2007 |
| 18. | South America | Bolivia | BO | 1 | - | 1 | 1999 |
| 19. | South America | Brazil | BR | 22 | - | 22 | 1990-2005 |
| 20. | South America | Chile | CL | - | 25 | 25 | - |
| 21. | South America | Colombia | CO | 27 | - | 27 | 2001-2006 |
| 22. | South America | Ecuador | EC | 5 | - | 5 | 1989-2006 |
| 23. | South America | Guyana | GY | 1 | - | 1 | 2000 |
| 24. | South America | Peru | PE | 1 | - | 1 | 2004 |
| 25. | South America | Suriname | SR | 4 | - | 4 | 2000 |
| 26. | South America | Uruguay | UY | 2 | - | 2 | 2001 |
| 27. | South America | Venezuela | VE | 32 | 10 | 42 | 2004-2008 |
|  | - | - |  | **263** | **50** | **313** | **1983-2009** |
