# Supplementary material for: Reviewing the History of HIV-1: Spread of Subtype B in the Americas
Source: PLoS One. 2011 Nov 23;6(11):e27489. doi: 10.1371/journal.pone.0027489 (PMC3223166; doi:10.1371/journal.pone.0027489)
Supplement: Table S2 — Percentage of sequences grouped within each of the four main clades inferred in the Bayesian phylogeny of HIV-1 subtype B using 263 sequences from 25 countries sampled in North America, Central America, Caribbean and South America. (DOC) [file pone.0027489.s002.doc]

**Table S2.** Number of sequences grouped within each clade of the Bayesian phylogeny of HIV-1 subtype B and the percentage of sequences from each sampled region, including 71 sequences from North America, 8 sequences from Central America, 80 sequences from the Caribbean, and 104 sequences from South America.

| **Clade (number of sequences)** | **Region** | **Number of sequences (% of the clade)** | **Number of sequences (% of the region)** |
| --- | --- | --- | --- |
| Caribbean Clade (51 sequences) | North America | 1/51 (1.96%) | 1/71 (1.41%) |
|  | Central America | 0/51 (0%) | 0/08 (0%) |
|  | Caribbean | 42/51 (82.35%) | 42/80 (52.5%) |
|  | South America | 8/51 (15.69%) | 8/104 (7.69%) |
| Pandemic Clade A (6 sequences) | North America | 1/06 (16.67%) | 1/71 (1.41%) |
|  | Central America | 0/06 (0%) | 0/08 (0%) |
|  | Caribbean | 1/06 (16.67%) | 1/80 (1.25%) |
|  | South America | 4/06 (66.66%) | 4/104 (3.85%) |
| Pandemic Clade B (90 sequences) | North America | 21/90 (23.33%) | 21/71 (29.58%) |
|  | Central America | 1/90 (1.11%) | 1/08 (12.5) |
|  | Caribbean | 25/90 (27.78%) | 25/80 (31.25%) |
|  | South America | 43/90 (47.78%) | 43/104 (41.35%) |
| Pandemic Clade C (116 sequences) | North America | 48/116 (41.38%) | 45/71 (67.6%) |
|  | Central America | 7/116 (6%) | 7/08 (87.5%) |
|  | Caribbean | 12/116 (10.35%) | 12/80 (15%) |
|  | South America | 49/116 (42.24%) | 49/104 (47.11%) |
